# Clinical value of folate receptor-positive circulating tumor cells in patients with esophageal squamous cell carcinomas: a retrospective study

**DOI:** 10.1186/s12885-023-11565-z

**Published:** 2023-11-30

**Authors:** Qiang Zhou, Qiao He, Wenwu He, Chenghao Wang, Guangyuan Liu, Kangning Wang, Haojun Li, Jialong Li, Wenguang Xiao, Qiang Fang, Lin Peng, Yongtao Han, Dongsheng Wang, Xuefeng Leng

**Affiliations:** 1 Department of Thoracic Surgery, Sichuan Clinical Research Center for Cancer, Sichuan Cancer Hospital & Institute, Sichuan Cancer Center, Affiliated Cancer Hospital of University of Electronic Science and Technology of China, No.55, Section 4, South Renmin Road, Chengdu, 610041 China; 2 Department of Clinical Laboratory, Sichuan Clinical Research Center for Cancer, Sichuan Cancer Hospital & Institute, Sichuan Cancer Center, Affiliated Cancer Hospital of University of Electronic Science and Technology of China, Chengdu, China

**Keywords:** Esophageal squamous cell carcinomas, Folate receptor, Circulating tumor cells, Overall survival, Disease-free survival

## Abstract

**Background:**

The aim of the study is to explore the role of preoperative folate receptor-positive circulating tumor cell (FR^+^CTC) levels in predicting disease-free survival (DFS) and overall survival (OS) in patients with esophageal squamous cell carcinomas (ESCC).

**Methods:**

Three ml blood samples were prospectively drawn from ESCC patients, and ligand-targeted polymerase chain reaction (LT-PCR) was used for the quantification of FR^+^CTCs. Other serum indicators were measured by traditional methods. Clinicopathological characteristics were obtained from the hospital medical record system, DFS and OS data were obtained by follow-up. The correlation between clinico-pathological characteristics, DFS, and OS and FR^+^CTCs were analyzed, respectively. Risk factors potentially affecting DFS and OS were explored by Cox regression analysis.

**Results:**

there were no significant correlations between FR^+^CTCs and patient age, sex, albumin, pre-albumin, C-reactive protein (CRP), ferritin and CRP/Albumin ratio, tumor size, grade of differentiation, lymph node metastasis, TNM stage, perineural invasion/vessel invasion (all *P* > 0.05). Nevertheless, preoperative FR^+^CTCs were an independent prognostic factor for DFS (HR 2.7; 95% CI 1.31-, *P* = 0.007) and OS (HR 3.37; 95% CI 1.06-, *P* = 0.04). DFS was significantly shorter for patients with post-operative FR^+^CTCs ≥ 17.42 FU/3ml compared with patients < 17.42 FU/3ml (*P* = 0.0012). For OS, it was shorter for patients with FR^+^CTCs ≥ 17.42 FU/3ml compared with patients < 17.42 FU/3ml, however, the difference did not reach statistical significance (*P* = 0.51).

**Conclusions:**

ESCC patients with high FR^+^CTCs tend to have a worse prognosis. FR^+^CTCs may monitor the recurrence of cancers in time, accurately assess patient prognosis, and guide clinical decision-making.

**Trial registration:**

The study was approved by the Sichuan Cancer Hospital & Institute Ethics Committee (No. SCCHEC-02-2022-050).

## Introduction

Esophageal cancer (EC) is the seventh most common cancer worldwide and the sixth leading cause of cancer death in 2020 [1]. EC was divided into two most common subtypes: esophageal squamous cell carcinomas (ESCC) and esophageal adenocarcinoma (EAC) according to histologic character, and the geographic variation in their incidence substantially differs. In China, EC cases constitute more than 50% of the global burden, and ESCC accounts for more than 90% of EC cases with a very high mortality rate [2]. Since the early symptoms of ESCC are often difficult to recognize, most of the patients are at an advanced stage when they were first diagnosed, which results in a poor prognosis, with a 5-year survival rate of only about 20% [3].

Early-stage EC is usually treated with endoscopy, in contrast, the conventional treatment of thoracic locally advanced disease is neo-adjuvant chemo-radiotherapy (NCRT) followed by surgical treatment, cervical esophageal cancers are generally treated with explicit chemo-radiation [4]. Accurate diagnosis and staging determine the treatment. However, the diagnosis and staging of EC is highly dependent on imaging or pathology. This makes it very difficult accurately assess the impact of micro-metastasis of tumor cells in circulatory system on patients’ treatment and prognosis.

Circulating Tumor Cells (CTCs) are tumor cells released from the primary neoplasm site and appear in the circulatory system as single cells or clusters, first described by Dr. Thomas Ashworth in 1869. CTCs shed and relocate at a new site, in a process often referred to as tumor metastasis [5]. In recent decades, the CTCs in peripheral blood have been demonstrated to be a diagnostic biomarker and prognostic factor and offer new strategies for treating cancer, especially lung cancer [6]. For ESCC patients, CTCs were explored as a biomarker for diagnosing, staging and evaluating treatment efficacy [7–10]. However, the CellSearch™ system is apparently ineffective in detecting CTCs in ESCC patients. For the CellSearch™ system, only epithelial marker-positive CTCs could be detected [7]. In Chen et al. study, mesenchymal CTCs are the reliable marker for assessing prognosis [8]. Folate receptor (FR) is highly expressed in various types of solid tumors [11]. It has been used as a biomarker for in vivo imaging and the therapeutic effect in patients with ovarian cancer (OC) [12]. Previous studies revealed that folate receptor-positive CTCs (FR^+^CTCs) have a high sensitivity of 72–78% and specificity of 82–90% for the diagnosis of lung cancer [13]. Recently, FR^+^CTCs have been proven to be an independent biomarker of prognosis in patients with lung cancer, which can predict the recurrence and metastasis of non-small cell lung cancer (NSCLC) [14]. Pre-operative FR^+^CTCs were an independent prognostic factor of relapse-free survival (RFS) in patients with NSCLC who underwent surgery. Further, overall survival (OS) was longer in patients with lower Pre-operative FR^+^CTC levels than those with higher Pre-operative FR^+^CTC levels. However, the difference was not statistically significant [15]. As far as we know, up to now, there is no study on the diagnosis and prognosis of FR^+^CTC in patients with ESCC. Thus, the diagnosis and predictive values of Pre-operative FR^+^CTCs level for ESCCs remain to be clarified.

The aim of our study was to investigate the predictive role of Pre-operative FR^+^CTC level on disease-free survival (DFS) and OS in patients with ESCC.

## Materials and methods

### Study design

This was a retrospective observational study. The inclusion criteria of the present study as follows: (1) Older than 18 years of age; (2) histological diagnosis of ESCC; (3) Pre-operative FR^+^CTC value and survival data were available. Patients with the following reasons were excluded: (1) a history of irrelevant carcinoma; (2) cervical esophageal cancer. From January 2018 to February 2021 and follow up to June 2022.

The study was approved by the Sichuan Cancer Hospital & Institute Ethics Committee (No. SCCHEC-02-2022-050).

### FR^+^ CTC analysis

CTCs were enriched and quantified using the CytoploRare Kit according to product manuals (Genosaber Biotech, Shanghai, China). In brief, 3 ml of peripheral blood were withdrawn into an EDTA-containing anti-coagulant vacuum tube from each patient one day before surgery and within one week after surgery. Lysis of erythrocytes, immuno-magnetic depletion of leukocytes from the whole blood to enrich CTCs. Then, as previously described, CTCs were labeled with specific probes (an oligonucleotide that is conjugated to the tumor-specific ligand folic acid). The FR^+^CTC level was quantified by ligand-targeted polymerase chain reaction (LT-PCR) [16, 17]. The LT-PCR reaction was performed under the following procedure: denaturation:95 °C, 2 min, annealing: 40 °C, 30 s, extension: 72 °C, 30 s, and then cooling at 8 °C for 5 min; 40 cycles of denaturation at 95 °C for 10 s, annealing at 35 °C for 30 s, and extension at 72 °C for 10 s. “FU” was a self-designated CTC unit which derived from a standard curve was used to denote the amount of FR^+^CTCs in 3 ml peripheral blood. A series of standards containing oligonucleotides (10^−14^ to 10^−9^ M, corresponding to 2 to 2 × 10 [5] CTC units/3ml blood) are used for FR^+^CTC quantification.

### Measurement of serum indicators

Serum albumin was detected by the bromocresol green (BCG) method, and ≤ 35 g /L was defined as hypoalbuminemia. Pre-albumin (PA) was analyzed on a fully automated Cobas 8000 biochemical analyzer (Roche Diagnostics, Indianapolis, USA) by collecting blood in a vacuum tube and centrifuging at 3500 rpm for 5 min. Serum ferritin was detected by chemiluminescence method with chemiluminescence instrument i4000 (Abbott Company, USA). For the measure of C-reactive protein (CRP) level, serum was collected 1 day before the operation. Serum CRP was measured using a CRP enzyme-linked immunosorbent assay (ELISA) kit (Westtang Corporation, Shanghai, China), the normal range is 0–5 mg/L. CRP concentration is considered positive when it exceeds 5 mg/L. The CRP (mg/L)-albumin (g/L) ratio (CRP/Alb).

### Data collection and follow up

Clinicopathological characteristics and patients’ survival outcomes, including age, sex, smoking, alcohol drinking, tumor size, histologic grade, perineural invasion, vessel invasion, TNM stages, the types of surgery, and the chemotherapy and radiotherapy received by the patients were collected in the medical record system of Sichuan Cancer Hospital & Institute Esophageal Cancer Case Management Database (SCCH-ECCM Database). Telephone follow-up was carried out if outpatient follow-up was not available. Follow-up was carried out with each patient until death or October 2022. DFS defined as the time from surgery to recurrence. Overall survival (OS) was defined as the time from surgery to death from any reason.

### Statistical analysis

Categorical data was expressed as numbers and percentages, while non-normally distributed continuous data are expressed as median interquartile range (IQR). Categorical data compared between groups by Fisher’s exact test. Continuous data was compared using the Kruskal-Wallis test or Mann-Whitney U test. Survival curves were calculated using the Kaplan-Meier method and comparisons were made using the log-rank test. Cox proportional hazard regression analysis was used to assess the risk factors potentially affecting the DFS or OS. Potentially significant covariates (*P* < 0.05) were indentified in the univariate analysis for subsequent multivariate analysis. A final model selection was performed using a backward stepwise selection process with the smallest akaike information criterion (AIC) [18]. All statistical analyses were performed using R 4.0.0 (R Foundation for Statistical Computing, Vienna, Austria). *P* < 0.05 was considered to be statistically significant.

## Results

### Baseline characteristics of enrolled patients

Among the 881 patients with ESCC, 203 patients had paired FR^+^CTC values both pre- and post-operative, only 123 patients with Pre-operative FR^+^CTC and survival data, and 50 patients with pre- and post-operative paired FR^+^CTC values and survival data. Finally, 123 patients with ESCC (from January 2018 to February 2021) with pre-operative FR^+^CTC and survival data were included for analysis. Among them, men account for 86%, and the mean age was 62.0. The results of smoking, alcohol drinking, albumin, pre-albumin, CRP, ferritin, FR^+^CTC, tumor size, histologic grade, perineural invasion, vessel invasion, TNM stages, types of surgery, chemotherapy regimen, radiotherapy dosage, DFS and OS were shown in Table 1.

**Table 1:** Baseline characteristics of enrolled patients

Characteristics (*N* = 123)	Values
**Sex (n, %)**	
Male	106 (86.2%)
Female	17 (13.8%)
**Age (years)**	
Median [Q1-Q3]	62.0 [55.0–67.0]
**Tumor size (cm)**	
Median [Q1-Q3]	2.80 [2.15-4.00]
Missing	35 (28.5%)
**Smoking history (n, %)**	
No	38 (30.9%)
Yes	85 (69.1%)
**Alcohol drinking (n, %)**	
No	43 (35.0%)
Yes	80 (65.0%)
**Histologic grade (n, %)**	
Well differentiated	54 (43.9%)
Moderately differentiated	40 (32.5%)
Poorly differentiated	29 (23.6%)
**Perineural invasion (n, %)**	
No	86 (69.9%)
Yes	37 (30.1%)
**Vessel invasion (n, %)**	
No	88 (71.5%)
Yes	35 (28.5%)
**T stage (n, %)**	
0	18 (15.7%)
is	2 (1.74%)
1	20 (17.4%)
2	16 (13.9%)
3	57 (49.6%)
4	2 (1.74%)
**N stage (n, %)**	
0	49 (42.6%)
1	44 (38.3%)
2	19 (16.5%)
3	3 (2.61%)
**TNM stage (n, %)**	
I	26 (22.6%)
II	32 (27.8%)
III	52 (45.2%)
IV	5 (4.4%)
**Pre-operation CTC (FU/3ml)**	
Median [Q1-Q3]	10.80 [9.13–14.40]
**Post-operation CTC (FU/3ml)**	
Median [Q1-Q3]	8.67 [6.95–12.20]
**Albumin (g/L)**	
Median [Q1-Q3]	37.9 [35.2–41.4]
**Pre-albumin (mg/L)**	
Median [Q1-Q3]	205 [173–256]
**CRP (mg/L)**	
Median [Q1-Q3]	3.93 [1.23–12.40]
**Ferritin (ng/mL)**	
Median [Q1-Q3]	146 [84–206]
**Surgery (n, %)**	
No	7 (5.7%)
R0	116 (94.3%)
**Chemotherapy (n, %)**	
No	14 (11.5%)
Paclitaxel + platinum drugs	90 (73.8%)
Docetaxe + platinum drugs	4 (3.3%)
Tegafur	12 (9.8%)
Irinotecan	1 (0.8%)
Specific medication unknown	1 (0.8%)
**Radiotherapy (n, %)**	
No	13 (10.7%)
< 45 Gy	79 (65.3%)
>=45 & < 50.4 Gy	18 (14.9%)
>= 50.4 Gy	11 (9.1%)
**DFS time (months)**	
Median [Q1-Q3]	25.1 [14.4–32.0]
**OS time (months)**	
Median [Q1-Q3]	26.5 [18.0-34.1]

### Association between pre-operative FR^+^CTCs and clinicopathological characteristics

In 123 patients with pre-operative FR^+^CTC values and survival data, the FR^+^CTCs were not significantly correlated with patient age, sex, albumin, pre-albumin, CRP, ferritin and CRP/Albumin ratio (all *P*>0.05). Moreover, FR^+^CTCs were not associated with tumor size, grade of differentiation, lymph node metastasis, TNM stages, perineural invasion or vessel invasion (all *P*>0.05) (Tables 2 and 3).

**Table 2:** Association between preoperative FR^ +^ CTCs and clinicopathological characteristics in enrolled patients

**Characteristics**	**N(%)**	**FR**^**+**^** CTC (FU/3ml, median, IQR)**	***P* value**
**Total**	123 (100.0)	10.78 (9.13–14.41)	
**Gender**			0.889
Male	106 (86.2)	10.60 [9.13–14.30]	
Female	17 (13.8)	11.30 [9.33–14.60]	
**Age(years)**			0.689
< 60	52 (42.3)	10.60 [8.74–14.70]	
>= 60	71 (57.7)	10.80[9.34–13.70]	
**Tumor size (cm)**			0.228
<= 3	53 (60.2)	10.30 [9.15–12.70]	
> 3	35 (39.8)	11.00 [9.67–14.50]	
**Histologic grade**			0.634
Well-differentiated	54 (43.9)	10.50 [8.80–14.50]	
Moderately differentiated	40 (32.5)	11.00 [9.68–13.40]	
Poorly differentiated	29 (23.6)	10.20 [9.03–15.50]	
**T stage**			0.365
0	18 (15.7)	9.89 [7.94–14.50]	
is	2 (1.7)	10.70 [10.30–11.10]	
1	20 (17.4)	9.97 [9.12–11.50]	
2	16 (13.9)	9.95 [9.19-12.00]	
3	57 (49.6)	11.60 [9.68–14.60]	
4	2 (1.7)	12.90 [10.90–15.00]	
**N stage**			0.917
0	49 (42.6)	10.80 [9.34–14.40]	
1	44 (38.3)	10.90 [9.26–13.30]	
2	19 (16.5)	10.30 [9.10–13.60]	
3	3 (2.6)	9.50 [8.08–12.30]	
**TNM stage**			0.680
I	26 (22.6)	10.30 [9.13-13.00]	
II	32 (27.8)	10.90 [9.61-14.00]	
III	52 (45.2)	11.00 [9.34–14.40]	
IV	5 (4.4)	9.50 [8.83-15.00]	
**Perineural invasion**			0.679
No	86 (69.9)	10.70 [9.09–14.30]	
Yes	37 (30.1)	11.00 [9.50–14.40]	
**Vessel invasion**			0.801
No	88 (71.5)	10.80 [9.14–14.20]	
Yes	35 (28.5)	10.20 [9.15–14.80]

**Table 3:** Correlation of pre-operation FR^ + ^CTC and other clinical indices

Clinical indices	N	r_s_	*P* value
**Albumin**	123	0.00	0.98
**Pre-albumin**	122	-0.03	0.78
**CRP**	103	0.14	0.24
**Ferritin**	83	0.19	0.13
**CRP/Albumin**	103	0.14	0.26

### Association between FR^+^CTCs level and survival time

**Fig. 1:**
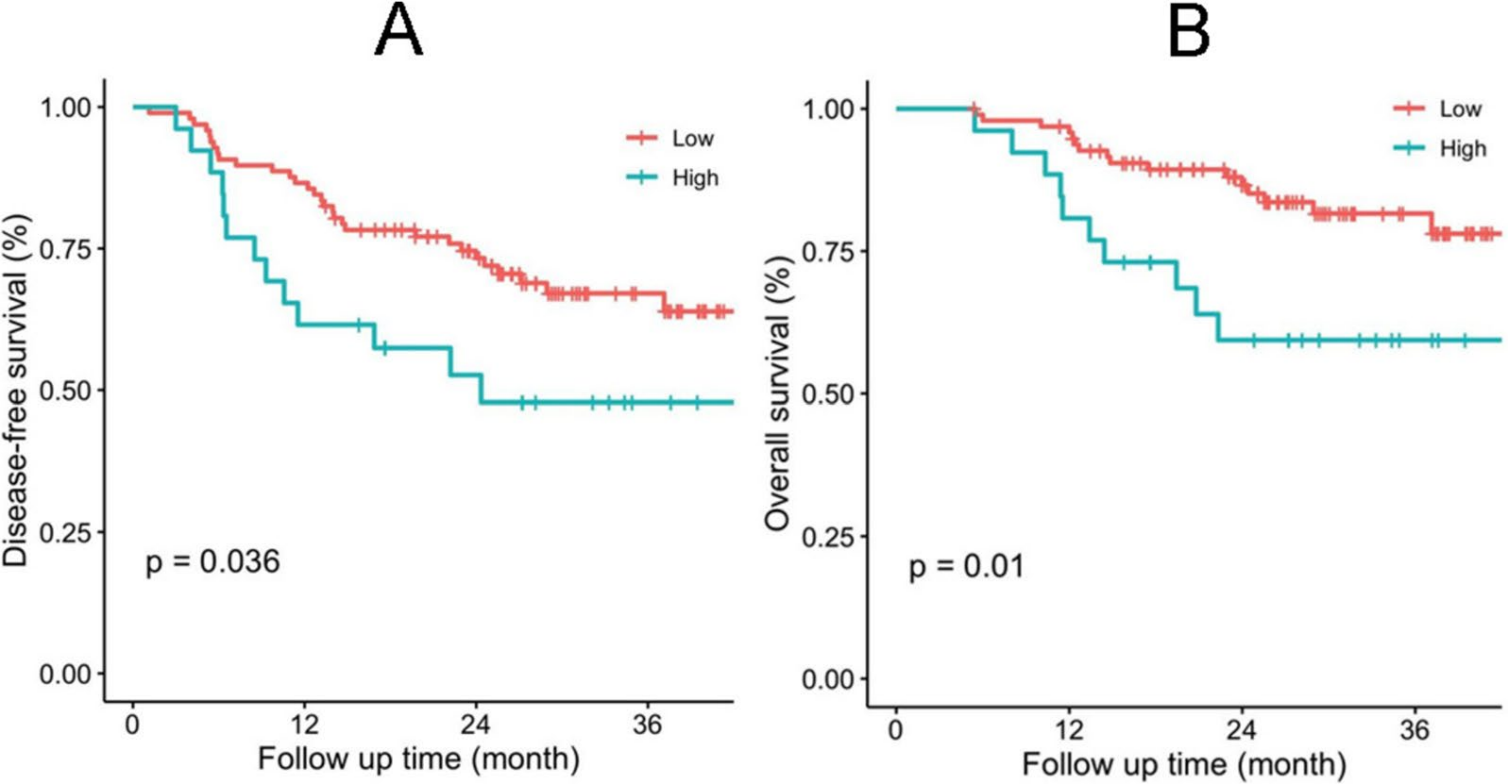
Kaplan-Meier curves of ESCC patients according to pre-operative FR^+^CTCs level. **A** Disease-free survival (DFS); (**B**) Overall survival (OS)

The median survival duration of the 123 patients was 26.5 months. The FR^+^CTCs level was associated with DFS. DFS was shorter for patients with FR^+^CTCs ≥ 14.79 FU/3ml compared with patients with FR^+^CTCs<14.79 FU/3ml (*P* = 0.036, Fig. 1A). In the 123 patients, the result of univariate Cox regression analyses indicated that smoking, alcohol consumption, pre-operative FR^+^CTC, post-operative FR^+^CTC, N stage, stage IV, albumin, and CRP were significantly associated with DFS (Table 4, all *P*<0.05), Further, pre-operative FR^+^CTCs ≥ 14.79 was an independent prognostic factor for DFS in the multivariate Cox regression analysis (HR, 95%CI 2.76, 1.31–5.8, *P* = 0.007) (Table 4). Moreover, alcohol consumption (HR, 95%CI: 2.37, 1.04-, *P* = 0.041), stage II (HR, 95%CI: 6.05, 1.333–27.54, *P* = 0.02), stage III (HR, 95%CI: 5.6, 1.29–24.29, *P* = 0.021), stage IV (HR, 95%CI 18.5, 2.97-116.05, *P* = 0.002) and CRP (HR, 95%CI: 2.24, 1.15-, *P* = 0.018) were independent prognostic factors for DFS, respectively (Table 4). OS was significantly shorter for patients with FR^+^CTCs ≥ 14.79 FU/3ml than those with FR^+^CTCs<14.79 FU/3ml (Fig. 1B, *P* = 0.01). The univariate Cox regression analyses showed that smoking, alcohol consumption, pre-operative FR^+^CTC, stage IV, albumin, and CRP were significantly associated with OS (all *P*<0.05, Table 5). Further, the multivariate Cox regression analysis showed that FR^+^CTCs ≥ 14.79 FU/3ml was an independent prognostic marker for OS (HR, 95%CI: 4.43, 1.72-, *P* = 0.002). Moreover, alcohol drinking (HR, 95%CI: 3.37, 1.06-, *P* = 0.04) and stage IV (HR, 95%CI: 40.09, 3.37-477.23, *P* = 0.003) were independent prognostic markers for OS (Table 5).

**Table 4:** Univariate and multivariate analyses of DFS

Characteristics	Events/N	Univariate analyses			Multivariate analyses		
		HR	95%CI	p	HR	95%CI	p
**Age**							
<= 60 years	17/52	-	-	-	-	-	-
> 60 years	26/71	1.1	0.60 - 2.03	0.762	-	-	-
**Sex**							
Female	3/17	-	-	-	-	-	-
Male	40/106	2.45	0.76 - 7.93	0.134	-	-	-
**Tumor size**							
<= 3 cm	20/53	-	-	-	-	-	-
>3 cm	14/35	1.04	0.52 - 2.06	0.911	-	-	-
**Smoking**							
No	7/38	-	-	-	-	-	-
Yes	36/85	2.66	1.18 - 5.97	0.018	-	-	-
**Alcohol drinking**							
No	9/43	-	-	-	-	-	-
Yes	34/80	2.34	1.12 - 4.88	0.024	2.37	1.04-5.42	0.041
**Differentiation degree**							
Well-differentiated	15/54	-	-	-	-	-	-
Moderately differentiated	15/40	1.23	0.60 - 2.52	0.567	-	-	-
Poorly differentiated	13/29	1.62	0.77 - 3.41	0.203	-	-	-
**Perineural invasion**							
No	26/86	-	-	-	-	-	-
Yes	17/37	1.73	0.85 - 3.52	0.130	-	-	-
**Vascular invasion**							
No	28/88	-	-	-	-	-	-
Yes	15/35	1.3	0.65 - 2.60	0.460	-	-	-
**Pre-operation FR**^**+**^**CTC (FU/3ml)**							
< 14.79	30/97	-	-	-	-	-	-
>= 14.79	13/26	1.98	1.03 - 3.81	0.040	2.76	1.31-5.80	0.007
**Post-operation FR**^**+**^**CTC (FU/3ml)**							
< 17.42	9/44	-	-	-	-	-	-
>= 17.42	4/6	5.92	1.74 - 20.13	0.004	-	-	-
**T**							
0	6/18	-	-	-	-	-	-
is	0/2	0	0 - Inf	0.996	-	-	-
1	6/20	0.81	0.26 - 2.53	0.721	-	-	-
2	3/16	0.53	0.13 - 2.13	0.375	-	-	-
3	26/57	1.5	0.62 - 3.65	0.371	-	-	-
4	1/2	2.79	0.33 - 23.3	0.343	-	-	-
**N**							
0	17/49	-	-	-	-	-	-
1	16/44	1.1	0.56 - 2.19	0.776	-	-	-
2	6/19	0.94	0.37 - 2.39	0.900	-	-	-
3	3/3	4.36	1.26 - 15.01	0.020	-	-	-
**TNM stage**							
I	6/26	-	-	-	-	-	-
II	13/32	2.07	0.78 - 5.44	0.142	6.05	1.33-27.54	0.02
III	19/52	1.86	0.74 - 4.67	0.184	5.6	1.29-24.29	0.021
IV	4/5	6.03	1.69 - 21.61	0.006	18.57	2.97-116.05	0.002
**Albumin (g/L)**							
>= 35	30/98	-	-	-	-	-	-
< 35	13/25	2.01	1.05 - 3.85	0.036	-	-	-
**Pre-albumin (mg/L)**							
>= 280	6/17	-	-	-	-	-	-
< 280	37/105	1.04	0.44 - 2.45	0.937	-	-	-
**CRP*(mg/L)***							
<= 8.2	18/65	-	-	-	-	-	-
> 8.2	19/38	2.41	1.26 - 4.61	0.008	2.24	1.15-4.38	0.018
**Ferritin (ng/mL)**							
<= 200	18/60	-	-	-	-	-	-
> 200	8/23	1.05	0.46 - 2.41	0.912	-	-	-
**Surgery**							
No	3/7	-	-	-	-	-	-
Yes	40/116	0.73	0.23 - 2.38	0.606	-	-	-
**Chemotherapy**							
No	6/14	-	-	-	-	-	-
Yes	37/108	0.92	0.39 - 2.20	0.856	-	-	-
**Radiotherapy**							
No	5/13	-	-	-	-	-	-
Yes	38/110	1.09	0.43 - 2.80	0.854	-	-	-

**Table 5:** Univariate and multivariate analyses of OS

Characteristics	Events/N	Univariate analyses			Multivariate analyses		
		HR	95%CI	p	HR	95%CI	p
**Age**							
<= 60 years	8/52	-	-	-	-	-	-
> 60 years	18/71	1.6	0.69 - 3.68	0.271	-	-	-
**Sex**							
Female	2/17	-	-	-	-	-	-
Male	24/106	2.07	0.49 - 8.75	0.324	-	-	-
**Tumor size**							
<= 3 cm	13/53	-	-	-	-	-	-
>3 cm	10/35	1.11	0.48 - 2.52	0.812	-	-	-
**Smoking**					-	-	-
No	3/38	-	-	-	-	-	-
Yes	23/85	3.72	1.12 - 12.40	0.032	-	-	-
**Alcohol drinking**							
No	4/43	-	-	-	-	-	-
Yes	22/80	3.31	1.14 - 9.61	0.028	3.37	1.06-10.74	0.04
**Differentiation degree**							
Well-differentiated	8/54	-	-	-	-	-	-
Moderately differentiated	10/40	1.61	0.63 - 4.08	0.319	-	-	-
Poorly differentiated	8/29	1.95	0.73 - 5.19	0.182	-	-	-
**Perineural invasion**							
No	10/47	-	-	-	-	-	-
Yes	11/37	1.56	0.66 - 3.67	0.312	-	-	-
**Vascular invasion**							
No	11/52	-	-	-	-	-	-
Yes	10/35	1.4	0.59 - 3.29	0.444	-	-	-
**Pre-operation FR**^**+**^**CTC (FU/3ml)**							
< 14.79	16/97	-	-	-	-	-	-
>= 14.79	10/26	2.71	1.23 - 5.99	0.013	4.43	1.72-11.4	0.002
**Post-operation FR**^**+**^**CTC (FU/3ml)**							
< 17.42	5/44	-	-	-	-	-	-
>= 17.42	1/6	2.05	0.23 - 18.50	0.521	-	-	-
**T**							
0	3/18	-	-	-			
is	0/2	0	0 - Inf	0.997			
1	6/20	1.61	0.40 - 6.44	0.503	-	-	-
2	1/16	0.38	0.04 - 3.67	0.405	-	-	-
3	15/57	1.69	0.49 - 5.84	0.408	-	-	-
4	1/2	5.26	0.54 - 51.01	0.152	-	-	-
**N**							
0	11/49	-	-	-	-	-	-
1	11/44	1.17	0.51 - 2.70	0.713	-	-	-
2	2/19	0.48	0.11 - 2.19	0.347	-	-	-
3	2/3	4.05	0.89 - 18.50	0.071	-	-	-
**TNM stage**							
I	3/26	-	-	-	-	-	-
II	10/32	3.15	0.87 - 11.46	0.082	8.13	0.98-67.37	0.052
III	10/52	1.97	0.54 - 7.17	0.302	5.88	0.74-46.75	0.094
IV	3/5	9.11	1.81 - 45.75	0.007	40.09	3.37-477.23	0.003
**Albumin (g/L)**							
>= 35	17/98	-	-	-	-	-	-
< 35	9/25	2.43	1.08 - 5.47	0.032	-	-	-
**Pre-albumin (mg/L)**							
>= 280	4/17	-	-	-	-	-	-
< 280	22/105	0.93	0.32 - 2.69	0.887	-	-	-
**CRP*(mg/L)***							
<= 8.2	10/65	-	-	-	-	-	-
> 8.2	12/38	2.54	1.09 - 5.88	0.030	2.21	0.89-5.47	0.088
**Ferritin (ng/mL)**							
<= 200	10/60	-	-	-	-	-	-
> 200	5/23	1.23	0.42 - 3.62	0.702	-	-	-
**Surgery**							
No	1/7	-	-	-	-	-	-
Yes	25/116	1.59	0.22 - 11.74	0.649	-	-	-
**Chemotherapy**							
No	6/14	-	-	-	-	-	-
Yes	20/108	0.49	0.20 - 1.23	0.129	-	-	-
**Radiotherapy**							
No	5/13	-	-	-	-	-	-
Yes	21/110	0.57	0.21 - 1.52	0.259	-	-	-

**Fig. 2:**
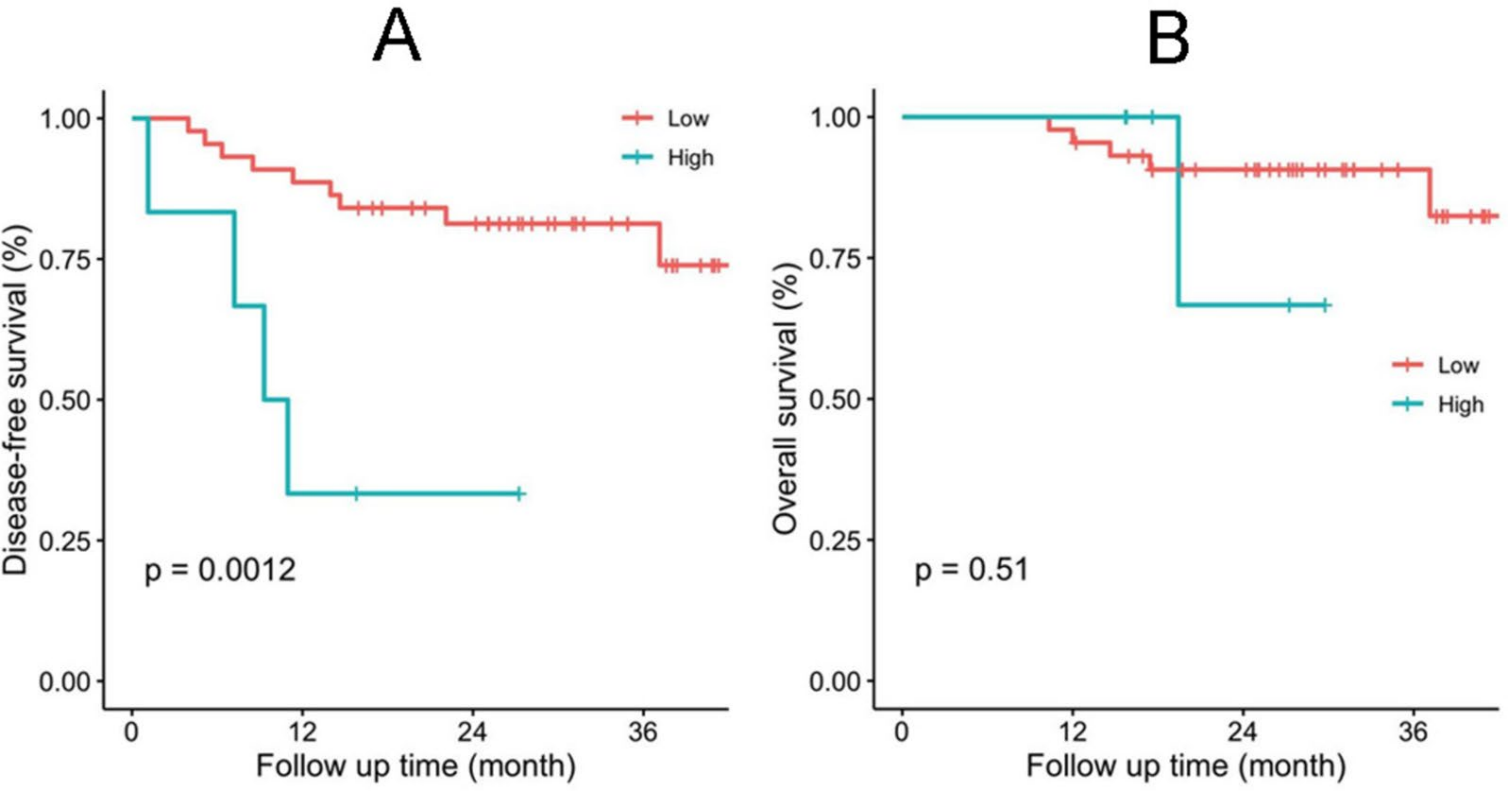
Kaplan-Meier curves of ESCC patients according to post-operative FR^+^CTCs level. **A** Disease-free survival (DFS); (**B**) Overall survival (OS)

As a subgroup, the 50 patients who had post-operative FR^+^CTC values were analyzed, and the results showed post-operative FR^+^CTC values were associated with DFS (Fig. 2A), DFS was significantly shorter for patients with FR^+^CTCs ≥ 17.42 FU/3ml compared with patients with FR^+^CTCs<17.42 FU/3ml (*P* = 0.0012). For OS, it was shorter for patients with FR^+^CTCs ≥ 17.42 FU/3ml compared with patients with FR^+^CTCs<17.42 FU/3ml, nonetheless, the difference was not statistically significant (Fig. 2B, *P* = 0.51).

## Discussion

In this study, LT-PCR technology was used in FR^+^CTCs detection in ESCC. The result suggested FR^+^CTC was not significantly related to patient’s age, sex, albumin, pre-albumin, CRP, ferritin and CRP/Albumin ratio. It was discovered to be connected to the patient’s DFS and OS, though. These findings indicate that FR^+^CTCs are independent predictors of survival in ESCC patients.

### Detection of FR^+^CTCs in ESCC patients

Over the past decade, numerous CTC enrichment and detection methods have developed, including density gradient centrifugation, immunomagnetic cell enrichment, flowcytometry, size-based filtration and isolation, PCR-based assays using a variety of chosen markers, and immunoassays against surface antigens [19–23]. The majority of these procedures are not used on a regular basis in clinical practice due to a lack of standardization, reproducibility, or assay duration, most of these techniques are not implemented in clinical routine. None has emerged as the benchmark for CTC testing [24]. To accurate CTC detection in metastatic breast, colon, and prostatic malignancies, only the CellSearch system has been received approval by the United States (U.S.) Food and Drug Administration (FDA). The CellSearch™ system, which focuses on detecting epithelial marker positive CTCs in ESCC patients, appears to be ineffective at doing so, there was no significant relationship between the presence of CTCs and any of the following patient characters: age, sex, median alcohol consumption, neutrophil/lymphocyte ratio, serum CEA, and WHO PS, even tumor location, size, depth, grade of differentiation, lymph node metastasis, or lymphatic or venous invasion was not significantly [7], which were consistent with our results. Age, sex, albumin, pre-albumin, CRP, ferritin, CRP/Albumin ratio, tumor size, differentiation grade, lymph node metastases, perineural invasion, or vascular invasion were not statistically related with FR ^+^ CTC in our study. The isolation and quantification of mesenchymal CTCs are significant for evaluating the pre-operative condition and treatment efficacy of ESCC patients in comparison to the previous work, which concentrated solely on epithelial marker-positive CTCs. And the findings revealed a statistically significant result between mesenchymal CTC count, ESCC clinical stage, and neoadjuvant chemotherapy effectiveness [8]. Immunomagnetic enrichment for CTCs that express epithelial cell adhesion molecules (EpCAM) using magnetic beads with antibody coatings, followed by fluorescent labeling, is the basis for detection [25]. The major problem of this approach is that it can only detect CTCs that still exhibit epithelial markers, rather than CTCs that undergone the epithelial-mesenchymal transition (EMT) [26]. Because CTCs may have already undergone an EMT process and display stem cell function. Therefore, they can provide false-negative results by upregulating their mesenchymal markers while downregulating their epithelial markers, enabling them to escape hematological detection [27].

### FR^+^CTCs are predictors of clinical outcomes of ESCC patients

The Isolation by Size of Epithelial Tumor cells (ISET)-based CTC assays can detect micro-metastases in ESCC patients that cannot be detected by traditional examination methods and may be used as prognostic indicators of disease progression and clinical outcomes in ESCC. The risk of disease progression was 3.8 fold higher in ESCC patients in whom CTCs > 2, compared with patients CTCs ≤ 2, and CTCs > 0 was an independent prognostic marker for tumor recurrence [9]. CTC-positive ESCC patients typically have a poorer prognosis, which indicates that timely monitoring of the recurrence and metastasis of ESCC may benefit from CTC detection [28]. The CTC quantity was demonstrated to be an independent prognostic factor in patients with unresectable ESCC, before concurrent chemoradiotherapy (CCRT) [29].

The China FDA (CFDA) have approved the use of FR^+^CTCs in clinical lung cancer detection [30]. Numerous earlier investigations have shown a connection between FR^+^CTCs and the prognosis of individuals with lung cancer [14, 15]. In patients with NSCLC, the risk of recurrence/metastasis was 5.49 times higher in the group with serum FR^+^CTC concentration ≥ 11 FU/3 ml in the group with FR^+^CTC concentration < 11 FU/3 ml [14]. Patients with low FR^ +^ CTC levels were linked with prolonged OS in a multivariate COX regression analysis; however, the conclusion did not achieve statistical significance. FR^ +^ CTC levels (cutoff: 7.9 FU/3 ml) were independent predictive variables of extended RFS [15]. According to a recent study FR is highly expressed in EC patients and serves as an independent prognostic factor in ESCC patients [31]. However, applying FR^+^CTCs in predicting the prognosis of EC requires further clarification. In our study, patients with FR^+^CTCs ≥ 14.79 FU/3ml in pre-operative blood had significantly shorter DFS and OS, suggesting that pre-operative FR^+^CTC may be a useful biomarker for DFS and OS of patients with ESCC, which agreed with the findings of earlier research [28, 29]. Stage IV cancer always indicates an advanced disease, according to our analysis, the stage IV variable has a strong correlation with DFS and OS. Metastasis occurs when tumor cells intrusive into the circulating system and become CTCs. CTC can act as disseminated tumor cells (DTC) to its new metastatic site after leaving the bloodstream. Several key factors determine the fate of DTC, such as the biological characteristics of CTC, and the tumor microenvironment (TME) in the metastatic niche [32]. The activation and proliferation of DTC can be caused by inflammatory stimuli [33], which happened in ESCC TME [34]. As to the biological characteristics, a study has found that the extracted CTCs contain many macrophages adhering to them. Further in vitro research discovered that CTCs obtained better mechanical adaptation phenotypes, with low hardness, adhesion, and deformation [35]. Furthermore, the other study focusing on primary and metastatic tumor ecosystems in ESCC revealed an accumulation of metastatic macrophages in metastatic ESCC lymph nodes [36]. These might be the underpinning mechanisms that could illustrate why esophageal cancer patients with high circulating tumor cell counts have poorer prognoses.

Additionally, in a preliminary study, elevated CRP level was linked to tumor progression and poor survival in patients with ESCC [37, 38]. For the low- and high-CRP groups in the ESCC, The overall 5-year survival rates were 90.6% and 63.6%, respectively, and the results were statistically significant (*p* = 0.043) [39]. High CRP is a predictive risk factor for DFS and OS in patients with ESCC, and the results of our study in the analysis of DFS in line with the previous finding. Even though the P value of multivariate analyses of CRP in OS is slightly higher than 0.05, which is 0.088, we still consider that it was significant in the real world, as we only have 43 events and could only include four variables in our model. CRP is the most important acute-phase protein. The association between cancer and inflammation is known for years, and the mechanisms behind CRP rise are well understood. Inflammation can be triggered by malignant tumor cells and vice versa. The research on TME of ESCC published on Nature Communication indicates that exhausted T cells, exhausted NK cells, regulatory T (Treg) cells, alternatively activated macrophages (M2), and tolerogenic dendritic cells (tDCs) were enriched in ESCC tissues, suggesting inflammation in TME in ESCC [34]. The hepatocytes’ produced CRP is known to be stimulated by a few proinflammatory cytokines, including interleukin-1, interleukin-6, tumor necrosis factor, interferon, and tumor growth factor. These cytokines also affect the survival, growth, mutation, proliferation, differentiation, and migration of tumor cells [40].Due to the above mechanism, pre-operative serum CRP levels constitute a significant predictive indicator in patients with various carcinomas [41, 42].

There were some inherent limitations in this study. One of them is the limitation of the retrospective nature; hence, a prospective study should be conducted to further explore the relationship between FR^+^CTC level and survival outcome. Furthermore, the predictive value of FR ^+^ CTC in ESCC needs to be explored in studies with larger sample sizes and longer follow-ups.

## Conclusions

Patients with FR^+^CTCs positive patients tend to have worse prognoses. We predict that CTCs may be used to rapidly monitor cancer recurrence and metastasis, precisely guided the clinical treatment and accurately assess patient prognosis and providing reference for timely adjustment of treatment strategy.

## Data Availability

The data that support the findings of this study are available from the corresponding author upon reasonable request.

## Abbreviations

AIC: Akaike information criterion
BCG: Bromocresol green
CFDA: China Food and Drug Administration
CRP: C-reactive protein
CTCs: Circulating Tumor Cells
DFS: Disease free survival
EAC: Esophageal adenocarcinoma
EC: Esophageal cancer
EpCAM: Epithelial cell adhesion molecules
ESCC: Esophageal squamous cell carcinomas
FR: Folate receptor
FR^+^CTC: Folate receptor-positive circulating tumor cell
LT-PCR: Ligand-targeted polymerase chain reaction
NCRT: Neo-adjuvant chemo-radiotherapy
NSCLC: Non-small cell lung cancer
OC: Ovarian cancer
OS: Overall survival
PFS: Progression free survival
RFS: Relapse-free survival
U.S.: United States
